# Cell Death in Chondrocytes, Osteoblasts, and Osteocytes

**DOI:** 10.3390/ijms17122045

**Published:** 2016-12-06

**Authors:** Toshihisa Komori

**Affiliations:** Department of Cell Biology, Unit of Basic Medical Sciences, Nagasaki University Graduate School of Biomedical Sciences, Nagasaki 852-8588, Japan; komorit@nagasaki-u.ac.jp; Tel.: +81-95-819-7630; Fax: +81-95-819-7633

**Keywords:** osteoarthritis, p53, Rb, ATP, DAMPs, Rankl, BCLXL, FoxO, osteoarthritis, apoptosis, necrosis

## Abstract

Cell death in skeletal component cells, including chondrocytes, osteoblasts, and osteocytes, plays roles in skeletal development, maintenance, and repair as well as in the pathogenesis of osteoarthritis and osteoporosis. Chondrocyte proliferation, differentiation, and apoptosis are important steps for endochondral ossification. Although the inactivation of *P53* and *RB* is involved in the pathogenesis of osteosarcomas, the deletion of *p53* and inactivation of Rb are insufficient to enhance chondrocyte proliferation, indicating the presence of multiple inhibitory mechanisms against sarcomagenesis in chondrocytes. The inflammatory processes induced by mechanical injury and chondrocyte death through the release of danger-associated molecular patterns (DAMPs) are involved in the pathogenesis of posttraumatic osteoarthritis. The overexpression of *BCLXL* increases bone volume with a normal structure and maintains bone during aging by inhibiting osteoblast apoptosis. p53 inhibits osteoblast proliferation and enhances osteoblast apoptosis, thereby reducing bone formation, but also exerts positive effects on osteoblast differentiation through the Akt–FoxOs pathway. Apoptotic osteocytes release ATP, which induces the receptor activator of nuclear factor κ-B ligand (Rankl) expression and osteoclastogenesis, from pannexin 1 channels. Osteocyte death ultimately results in necrosis; DAMPs are released to the bone surface and promote the production of proinflammatory cytokines, which induce Rankl expression, and osteoclastogenesis is further enhanced.

## 1. Chondrocyte Death

### 1.1. Chondrocyte Proliferation and Apoptosis

#### 1.1.1. Runx2, Indian Hedgehog (Ihh), and Parathyroid Hormone-Related Peptide (Pthrp) Regulate Chondrocyte Proliferation and Differentiation

The growth plate is composed of reservoir, proliferating, prehypertrophic, hypertrophic, and terminal hypertrophic chondrocytes. The expression of Runx2 is up-regulated in prehypertrophic chondrocytes, and chondrocyte hypertrophy is mainly regulated by Runx2 and, to a lesser extent, by Runx3. Runx2 induces the expression of *Ihh*, which is also strongly expressed in prehypertrophic chondrocytes [1,2]. Ihh enhances chondrocyte proliferation [3]. It also induces the expression of Pthrp, which inhibits *Runx2* expression and chondrocyte hypertrophy through the protein kinase A (PKA) signaling pathway in a negative feedback loop [4,5,6]. Vascular invasion occurs at the layer of terminal hypertrophic chondrocytes. The preosteoblasts surrounding cartilage invade the layer of terminal hypertrophic chondrocytes with blood vessels. The terminal hypertrophic chondrocyte layer is invaded by osteoclasts, and preosteoblasts attach to the invaded surface of the terminal hypertrophic chondrocyte layer and become osteoblasts, which produce bone matrix proteins. Runx2 enhances vascular invasion and cartilage matrix degradation through the induction of *Vegf* and *Mmp13*, respectively, in terminal hypertrophic chondrocytes [7,8,9]. Terminal hypertrophic chondrocytes gradually die by apoptosis or become osteoblasts, and the terminal hypertrophic chondrocyte layer is replaced with bone [10,11]. Therefore, chondrocyte apoptosis is observed in the terminal hypertrophic chondrocyte layer, but is mostly absent in the other layers under physiological conditions.

#### 1.1.2. Cyclin D1 Plays an Important Role in Chondrocyte Proliferation

The progression of the cell cycle is controlled by the sequential synthesis of individual cyclins. Cyclin D activates Cdk4 and Cdk6 in the G1 phase, cyclin E activates Cdk2 in the G1/S phase, cyclin A activates Cdk2 and Cdk1 in the S phase, and cyclin B activates Cdk1 in the M phase [12]. Cyclin D1 is known to be regulated by numerous extracellular growth factors and hormones, including Pthrp, transforming growth factor β (TGFβ), Ihh, and Wnt5b, during chondrocyte proliferation [13,14,15]. *Cyclin D1* is also a well-established human oncogene because the amplification and overexpression of *Cyclin D1* are involved in many kinds of cancers [16]. In the growth plate, Cyclin D1 is strongly expressed in the proliferating layer [15], and *Cyclin D1*-deficient mice show dwarfism with a diminished proliferating layer in the growth plate [13,15,17,18]. Cdk4 or Cdk6 activated by Cyclin Ds phosphorylates Rb family proteins (pRb, p130, and p110), resulting in the release of E2f transcription factors. This leads to the transcriptional activation of E2f-responsive genes that are essential for DNA synthesis, including cyclins E and A [16]. Rb family proteins play an important role in chondrocyte proliferation and differentiation [19,20].

#### 1.1.3. Can the Overexpression of Both *Cdk6* and *Cyclin D1* Induce Chondrocyte Proliferation?

We examined the functions of Cdk6 and Cyclin D1 in chondrocyte proliferation and differentiation by generating transgenic mice overexpressing *Cdk6*, *Cyclin D1*, or both *Cdk*6 and *Cyclin D1* under the control of the *Col2a1* promoter, which directs transgene expression to chondrocytes [21]. *Cdk6* or *Cyclin D1* single transgenic embryos show normal phenotypes, whereas *Cdk6*/*Cyclin D1* double transgenic mice show dwarfism, retarded chondrocyte maturation, the increased incorporation of BrdU, and markedly enhanced chondrocyte apoptosis. Although more chondrocytes in *Cdk6*/*Cyclin D1* double transgenic mice enter the S phase, many fail to complete the cell cycle and die by apoptosis, indicating that the overexpression of both *Cdk6* and *Cyclin D1* is insufficient to enhance chondrocyte proliferation, but successfully induces apoptosis. However, many in vitro and in vivo studies have shown that the overexpression of *Cyclin D1* enhances cell proliferation, and Cyclin D1 plays an important oncogenic role in many cancers [16,21,22,23]. Rb is highly phosphorylated, while unphosphorylated p107 levels are elevated in *Cdk6*/*Cyclin D1* double transgenic mice [21]. *p107* is a target gene of E2f, and *p107* is also up-regulated in *Rb*^−/−^ and *p130*^−/−^ mouse embryonic fibroblasts [24,25,26]. Furthermore, the expression of the E2f target gene *Cdc6* is up-regulated, whereas that of E2f target genes, including *Cyclin E*, *Dihydrofolate reductase* (*Dhfr*), *Cdc25a*, and *B-Myb*, is down-regulated in *Cdk6*/*Cyclin D1* double transgenic mice [21]. The introduction of siRNA for *p107* reverses the expression of these down-regulated E2f target genes. The deletion of *p53* almost completely rescues chondrocyte apoptosis, but fails to enhance chondrocyte proliferation in *Cdk6*/*Cyclin D1* double transgenic mice. These findings indicate that the overexpression of *Cdk6*/*Cyclin D1* enhances G1/S cell cycle transition by phosphorylating Rb, and also that chondrocytes fail to complete their cell cycle and undergo p53-dependent apoptosis through the dysregulation of E2f target genes due to the up-regulation of *p107* [21]. Therefore, the deletion of *p53* in addition to the inactivation of Rb is insufficient to enhance chondrocyte proliferation, indicating the presence of multiple inhibitory mechanisms against sarcomagenesis in chondrocytes [21].

#### 1.1.4. p107 Plays a Key Role in Chondrocyte Proliferation and Apoptosis

*p107*^−/−^ mice show the most severe inhibition of chondrocyte differentiation among the knockout mice of Rb family genes. The inhibition of chondrocyte differentiation is further enhanced in *p107*^−/−^*p130*^−/−^ or *p107*^−/−^*Rb*^−/−^ mice and chondrocyte proliferation also increases without elevations in chondrocyte apoptosis [19,20,27]. Therefore, the phenotypic differences that exist between *p107*^−/−^*p130*^−/−^ and *p107*^−/−^*Rb*^−/−^ mice and *Cdk6/Ccnd1* double transgenic mice are enhanced chondrocyte proliferation and the lack of chondrocyte apoptosis in *p107*^−/−^*p130*^−/−^ and *p107*^−/−^*Rb*^−/−^ mice. When *Cdk6* and *Cyclin D1* are overexpressed, Rb, p107, and p130 are expected to be phosphorylated, leading to the release of repressing E2fs (E2f4, 5) and activation of activating E2fs (E2f1–3), both of which enhance the transcription of E2f target genes including *p107*. Therefore, the phosphorylation of Rb, p107, and p130 enhances the transcription of *p107* by E2f, and unphosphorylated p107 is continuously supplied. Under physiological conditions, unphosphorylated p107 associates with repressing E2fs, whereas unphosphorylated p107 also associates with activating E2fs when p107 is up-regulated, resulting in the dysregulation of E2f target gene expression, as reported in *Cdk6*/*Cyclin D1* double transgenic mice [28,29]. Therefore, p107 is a key regulatory molecule in chondrocyte proliferation and apoptosis.

### 1.2. Chondrocyte Death in Osteoarthritis (OA)

#### 1.2.1. Is Chondrocyte Death Involved in the Pathogenesis of OA?

OA is a common degenerative joint disease that is characterized by the progressive breakdown of articular cartilage in addition to changes in other joint components including the subchondral bone, menisci, synovium, ligaments, capsule, and muscles. A number of processes, including chondrocyte hypertrophy, inflammatory processes, and chondrocyte death, are involved in the pathogenesis of OA [9,30,31]. Apoptosis, autophagic cell death, and necrosis have been detected in OA cartilage [32]. If cells die through apoptosis or autophagy, death is completed by the removal of cells through engulfment by scavengers. In these cases, the integrity of cytoplasmic membranes is maintained when phagocytosis occurs. Although chondrocytes are isolated by an extracellular matrix, similar to osteocytes, as described later, the terminal phase of apoptosis or autophagic cell death is not engulfed by phagocytes, a transition to necrosis ensues, and cells are eliminated by cell disruption; this process is called secondary necrosis [33]. Therefore, the mixed morphology of apoptosis, autophagic cell death, and necrosis has been reported in OA cartilage [34]. Similar to osteocytes, autophagy also protects chondrocytes from apoptosis, and its failure leads to apoptosis [35,36].

Previous studies reported positive correlations between chondrocyte death by apoptosis and the severity of OA [37,38,39,40,41,42,43]. Furthermore, a significant decrease has been reported in chondrocyte numbers in articular cartilage with aging [39,44,45]. The intra-articular administration of caspase inhibitors has been shown to inhibit chondrocyte death and cartilage degradation in OA rabbit models [46,47]. In contrast, a recent study using a 3D confocal cartilage imaging technique showed that chondrocyte death by diphtheria toxin did not result in cartilage damage [48]. Therefore, it currently remains controversial whether chondrocyte death itself causes cartilage degradation.

#### 1.2.2. Chondrocyte Death during Mechanical Injury May Contribute to the Development of OA

**Figure 1:**
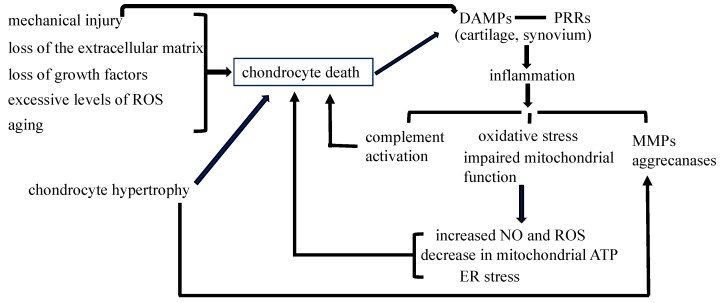
Chondrocyte death in osteoarthritis. Mechanical injury, the loss of the extracellular matrix, loss of growth factors, and excessive levels of reactive oxygen species (ROS) induce chondrocyte death in articular cartilage. Chondrocyte hypertrophy in articular cartilage induces the destruction of the cartilage matrix through the induction of matrix metalloproteinases (MMPs) and aggrecanases, and leads to chondrocyte apoptosis. The number of dead chondrocytes also increases in articular cartilage during aging. DAMPs (danger-associated molecular patterns) released by mechanical injury and chondrocyte secondary necrosis initiate non-infectious inflammatory responses through PRRs (pattern recognition receptors) expressed in osteoarthritis (OA) cartilage and the synovium, causing complement activation, oxidative stress, impaired mitochondrial function, and the induction of MMPs and aggrecanases. Complement activation, an increase in nitric oxide (NO) and ROS, a decrease in mitochondrial ATP, and endoplasmic reticulum (ER) stress further enhance chondrocyte death, resulting in the progression of osteoarthritis. The contribution of chondrocyte death to inflammatory processes needs to be evaluated. The inflammatory processes induced by mechanical injury contribute to the development of posttraumatic OA; however, their significance in non-posttraumatic OA remains unclear.

Chondrocyte death may be induced by mechanical injury, the loss of the extracellular matrix, loss of growth factors, or excessive levels of reactive oxygen species (ROS) [49] (Figure 1). Abnormal mechanical loading is a major risk factor for the development of OA. Injurious mechanical loading on articular cartilage causes collagen degradation, the loss of glycosaminoglycan (GAG), and chondrocyte apoptosis [50,51,52]. The importance of the extracellular matrix for chondrocyte survival has been demonstrated in integrin α1 knockout mice, which showed an increase in the number of apoptotic chondrocytes because integrins function as receptors that connect extracellular matrix proteins to various intracellular cytoskeletal proteins [53]. *Col2a1*-deficient mice also show an increase in the number of apoptotic chondrocytes in articular cartilage [54].

Mechanical injury increases danger-associated molecular patterns (DAMPs), including released and degraded cartilage matrix constituents, S100 family molecules, the high-mobility group box 1 (HMGB1) protein, purine metabolites, heat-shock proteins, and uric acid, which initiate non-infectious inflammatory responses through various pattern recognition receptors (PRRs) expressed in OA cartilage and the synovium, including toll-like receptor 2 (TLR2), TLR4, and the receptor for advanced glycation end-products (RAGE) [30]. Chondrocyte apoptosis or autophagic death also increases DAMPs because both result in secondary necrosis [33]. However, the significance of the DAMPs released from necrotic chondrocytes still needs to be investigated because the ablation of chondrocytes by diphtheria toxin prior to the operation for surgical OA reduces cartilage damage [48]. In this case, DAMPs must be considered to have already been released from necrotic chondrocytes and cartilage may contain no sufficient DAMPs when mechanical injury occurs.

#### 1.2.3. Non-Infectious Inflammatory Processes through PRRs Are Involved in the Pathogenesis of Posttraumatic OA

The signaling pathway through PRRs induces synovitis, transduces inflammation, increases the expression of matrix metalloproteinases (MMPs) and aggrecanases, and induces oxidative stress and cell death [55,56]. Inflammatory signals increase oxidative stress and impair mitochondrial function, an increase in nitric oxide (NO) and ROS induces chondrocyte apoptosis, and a decrease in mitochondrial ATP generation contributes to chondrocyte death [57,58,59,60,61]. Complement may be activated by DAMPs, and activated complement components may induce chondrocyte death [62,63]. Furthermore, oxidative stress and mitochondrial dysfunction cause endoplasmic reticulum (ER) stress, and the unsuccessful resolution of ER stress by the unfolded protein response (UPR) promotes chondrocyte apoptosis via Chop [64] (Figure 1). Adenosine monophosphate-activated protein kinase (AMPK) activity regulates energy metabolism via downstream mediators including the NAD^+^-dependent protein deacetylase SIRT1, and SIRT1 inhibits chondrocyte apoptosis [65,66,67]. Chondrocyte apoptosis increases during aging, and may be a base for the onset of OA. Inflammatory processes promote chondrocyte apoptosis during the progression of OA (Figure 1). However, the contribution of chondrocyte death to inflammatory processes needs to be further clarified. Although inflammatory processes contribute to the development of posttraumatic OA, their significance in the pathogenesis of non-posttraumatic OA remains to be evaluated.

## 2. Osteoblast Death

### 2.1. Roles of Apoptosis in the Regulation of Bone Mass and Osteoblast Differentiation

#### Osteoblast Apoptosis Regulates Bone Mass

Osteoblast apoptosis plays an important role in bone development and maintenance. Between 60% and 80% of the osteoblasts that originally assemble at the resorption pit have been estimated to die by apoptosis. Furthermore, bone loss caused by a sex steroid deficiency, glucocorticoid (GC) excess, or aging has been partly attributed to apoptosis in osteoblasts and osteocytes, and parathyroid hormone (PTH), bisphosphonate, and calcitonin exert anabolic effects on bone by inhibiting osteoblast and osteocyte apoptosis [68,69,70,71,72,73,74,75]. In GC excess, autophagy appears to protect osteocytes from the negative effects of GCs; however, when these effects reach a certain threshold, the process of autophagy induces apoptosis in cells. Excess GCs increase the levels of the pro-apoptotic factors, Bim and Bak; decrease that of the pro-survival factor, BclXL; up-regulate the expression of the p53 protein; increase ROS levels; inhibit Akt; and enhance ER stress [35].

The overexpression of *BCLXL* inhibits osteoblast apoptosis and increases the mineral density and trabecular and cortical bone volumes of femurs as well as the cancellous bone volume of vertebrae due to enhanced bone formation. The bone structure is similar to, but stronger than that in wild-type mice and the increased bone mass is maintained during aging in both sexes [76]. The deletion of *Bak* and *Bax* in osteoblasts also increases the mineral density and trabecular bone volume of femurs, whereas the cortical bone volume of femurs and cancellous bone volume of vertebrae are not increased and cortical bone shows severe porosity in aged mice [77]. Since the expression of *Rankl* and *Vegf* is up-regulated in aged mice, osteoblast maturation may be inhibited or osteocyte death may occur. Furthermore, the deletion of *Bak* and *Bax* in osteoblasts appears to be insufficient for the efficient inhibition of osteoblast apoptosis.

### 2.2. Osteoblast Apoptosis and Osteoblast Differentiation

#### 2.2.1. Osteoblast Apoptosis Suppresses Osteoblast Differentiation In Vitro by Reducing Cell Density during Cultivation

Our group and other groups described skeletal development in *Bcl2*^−/−^ mice and the differentiation and functions of *Bcl2*^−/−^ osteoblasts and osteoclasts [78,79,80]. *Bcl2*^−/−^ mice show an increased bone mass due to enhanced apoptosis in and the impaired differentiation of osteoclasts. However, the viability and differentiation of *Bcl2*^−/−^ osteoblasts are controversial in these studies. In vitro osteoblast cultures revealed that osteoblast apoptosis occurred similarly in *Bcl2*^−/−^ and wild-type osteoblasts or was enhanced in *Bcl2*^−/−^ osteoblasts [78,79]. In vitro osteoblast cultures also showed that the differentiation of *Bcl2*^−/−^ osteoblasts was similar to or impaired more than that of wild-type osteoblasts [78,79]. In our analyses, osteoblast apoptosis was found to be increased in *Bcl2*^−/−^ mice in vivo and *Bcl2*^−/−^ osteoblasts in vitro. Furthermore, in situ hybridization and real-time RT-PCR analyses have shown that osteoblast marker gene expression is increased in *Bcl2*^−/−^ mice, and in vitro osteoblastogenesis is also accelerated in *Bcl2*^−/−^ osteoblasts [80]. This discrepancy in osteoblast differentiation in *Bcl2*^−/−^ mice has been attributed to the different findings obtained on osteoblastogenesis in vitro. When primary osteoblasts were seeded at a concentration of 2.5 × 10^4^/cm^2^, osteoblast differentiation occurred similarly in wild-type and *Bcl2*^−/−^ osteoblasts. However, when primary osteoblasts were seeded at a higher concentration of 1 × 10^5^/cm^2^, osteoblast differentiation was greater in *Bcl2*^−/−^ osteoblasts than in wild-type osteoblasts [80]. Osteoblastogenesis in *BCL2*-overexpressing primary osteoblasts in vitro is also controversial, and was previously reported to be enhanced and inhibited [81]. The inhibition of osteoblastogenesis in *BCL2*-overexpressing primary osteoblasts is also dependent on the cell density seeded because the overexpression of *BCL2* enhances osteoblast differentiation by increasing cell density through the inhibition of apoptosis in vitro [81]. In accordance with in vitro findings, osteoblast differentiation is inhibited in osteoblast-specific *BCL2* transgenic mice [81]. This is in contrast to *Bcl2*^−/−^ osteoblasts for which osteoblast apoptosis was found to be enhanced during cultivation [80]. Therefore, the discrepancy in the differentiation of *Bcl2*^−/−^ osteoblasts is explained by a reduced cell density during cultivation, which decelerates osteoblast differentiation, due to increases in apoptosis in *Bcl2*^−/−^ osteoblasts.

#### 2.2.2. Apoptotic Responses Enhance Osteoblast Differentiation through the p53–Akt–FoxO Pathway

**Figure 2:**
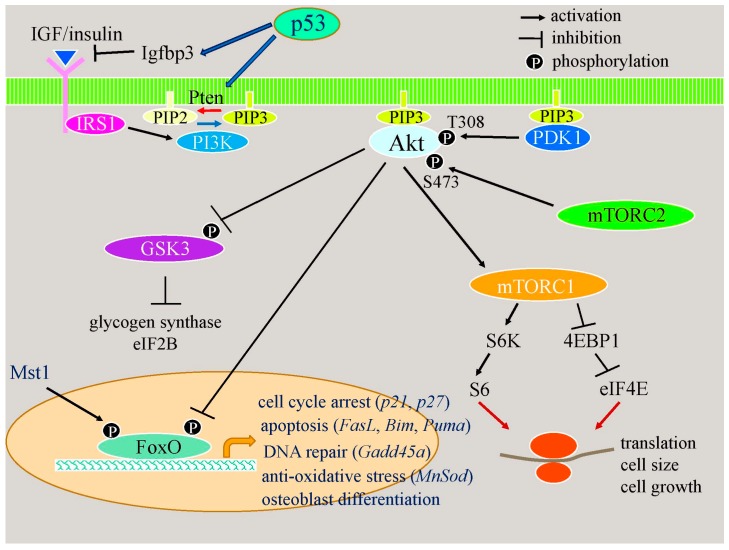
Acceleration of osteoblast differentiation through the p53–Akt–FoxO pathway. p53 induces the expression of *Igfbp3* and *Pten*. Igfbp3 inhibits IGF/insulin binding to the receptor, and Pten dephosphorylates PIP3 to PIP2. PIP3 recruits Akt to the cell membrane and Akt is activated by PDK1 and mTORC2. The decrease in PIP3 inactivates Akt. Akt regulates metabolism, cell growth, cell survival, cell proliferation, and cell differentiation through GSK3, mTORC1, and FoxOs. Akt phosphorylates FoxOs to inactivate them. FoxOs are involved in cell cycle arrest, apoptosis, DNA repair, anti-oxidative stress, and osteoblast differentiation.

Enhanced osteoblast differentiation in *Bcl2*^−/−^ mice is partly explained by the induction and activation of FoxOs [80]. FoxO1 and FoxO3a induce osteoblast differentiation [80,82,83,84]. FoxO proteins are inactivated through the phosphorylation of Thr24, S256, and S319 in FoxO1 and that of Thr32, S253, and S315 in FoxO3 by Akt, and are activated through the phosphorylation of S212 in FoxO1 and S207 in FoxO3a by Mst1, and Akt and Mst1 are activated by phosphorylation [85] (Figure 2). In *Bcl2*^−/−^ calvariae, FoxO1 and FoxO3a mRNA expression is up-regulated, the phosphorylation of THr24 in FoxO1 and Thr32 in FoxO3a is reduced, the phosphorylation of S207 in FoxO3a is mildly reduced, and the expression of FoxO target genes, including *FasL*, *Gadd45a*, and *Bim*, is up-regulated [80]. In accordance with these findings, the phosphorylation of Akt is severely reduced and that of Mst1 is mildly reduced. Therefore, FoxOs are activated through the inactivation of Akt in *Bcl2*^−/−^ calvariae [80] (Figure 2). *p53* mRNA and protein expression is up-regulated, and the expression of its target genes—*Pten* and *Igfbp3*—is also increased in *Bcl2*^−/−^ calvariae [80]. Thus, the activation of Akt is inhibited, at least in part, by the p53-dependent inactivation of Akt, leading to the activation of FoxOs (Figure 2). These findings indicate that apoptotic responses enhance osteoblast differentiation through the p53–Akt–FoxO pathway.

#### 2.2.3. p53 Reduces Bone Formation by Inhibiting Osteoblast Proliferation and Enhancing Osteoblast Apoptosis

Mice with activated mutations in *p53* exhibit osteopenia [86]. Furthermore, conditional knockout mice of *Mdm2*, which negatively regulates p53 activity, using the 3.6-kb *Col1a1* promoter in Cre transgenic mice resulted in reduced bone formation, and the differentiation of primary osteoblasts was inhibited in vitro [87]. The phenotypes of *p53*-deficient mice are controversial, with bone mass and bone formation being normal or increased [88,89,90]. The introduction of *p53* into wild-type and *p53*^−/−^ osteoblasts inhibits osteoblast differentiation [90]. However, the expression levels of p53 and osteocalcin have also been reported to be positively related [91]. Since the deletion of p53 enhances proliferation and inhibits apoptosis, the deletion of p53 may increase cell density in cultures, leading to the acceleration of osteoblast differentiation in vitro because osteoblast differentiation is dependent on cell density in vitro [76,80,81]. We found that skeletal development and chondrocyte differentiation in *p53*^−/−^ embryos were similar to those in wild-type embryos, whereas primary chondrocytes from *p53*^−/−^ embryos differentiated more rapidly than those from wild-type embryos in micromass cultures due to increased cell density (unpublished observation). The discrepancy in chondrocyte differentiation in vivo and in vitro is explained by chondrocyte proliferation being increased in *p53*^−/−^ chondrocytes in vitro, but not in the growth plate of *p53*^−/−^ mice, which demonstrates that the deletion of *p53* is insufficient to increase chondrocyte proliferation in vivo [21]. An increase in osteoblast numbers due to enhanced proliferation and reduced apoptosis may also lead to an increase in bone formation in *p53*^−/−^ mice, as reported previously [90]. Therefore, a major function of p53 in bone formation is to inhibit the proliferation of and enhance apoptosis in osteoblasts, leading to reduced bone formation. p53 also has a positive effect on osteoblast differentiation. p53 and FoxOs have similar functions, including inhibition of the cell cycle, DNA repair, and the induction of cell death, and have many overlapping target genes, such as *p21*, *Wip1*, *Gadd45a*, *Pa26*, and *Puma* [92]. The inhibition of osteoblast proliferation in *Bcl2*^−/−^ mice is, at least in part, due to the up-regulation of p53 and FoxOs [80].

## 3. Osteocyte Death

### 3.1. Osteocyte Death and Bone Remodeling

#### 3.1.1. ATP Released from Apoptotic Cells through Pannexin Channels Enhances Bone Resorption

**Figure 3:**
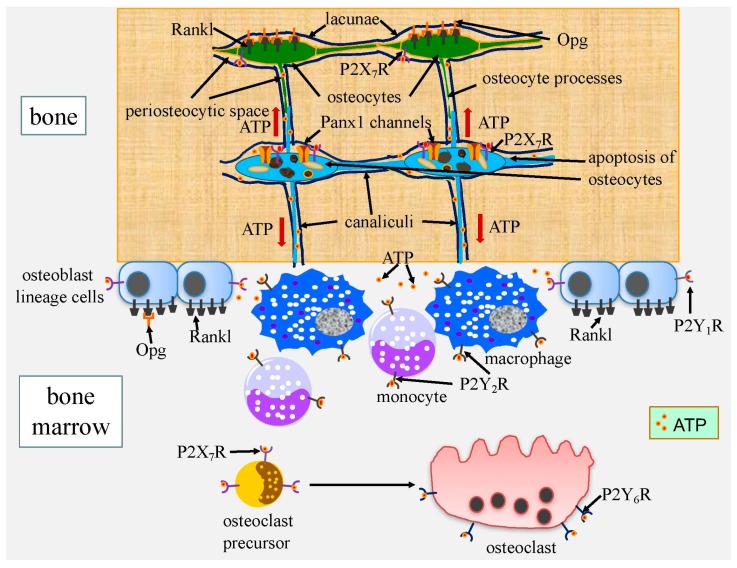
Induction of bone resorption by ATP released from apoptotic osteocytes. In apoptotic osteocytes, active Panx1 channels, which release ATP, are formed by the caspase-mediated cleavage of the C terminus. P2X_7_ receptor (P2X_7_R), which is activated by extracellular ATP, forms a complex with Panx1 and enhances ATP release. Extracellular ATP recruits macrophages and monocytes through P2Y_2_R, increases nuclear factor κ-B ligand (Rankl) expression in osteocytes through P2X_7_R and in osteoblasts through P2Y_1_R, increases osteoclast survival through P2Y_6_R, and enhances the membrane fusion of osteoclast precursor cells to form multinucleated osteoclasts through P2X_7_R. The up-regulation of Rankl in osteocytes will reduce the release of osteoprotegerin (Opg), which is secreted by osteocytes, to the bone surface. It has been shown that Panx1 and P2X_7_R are required for Rankl up-regulation in osteocytes in fatigued bone. However, the functions of ATP released from apoptotic osteocytes shown here have not yet been directly proven in damaged bone.

**Figure 4:**
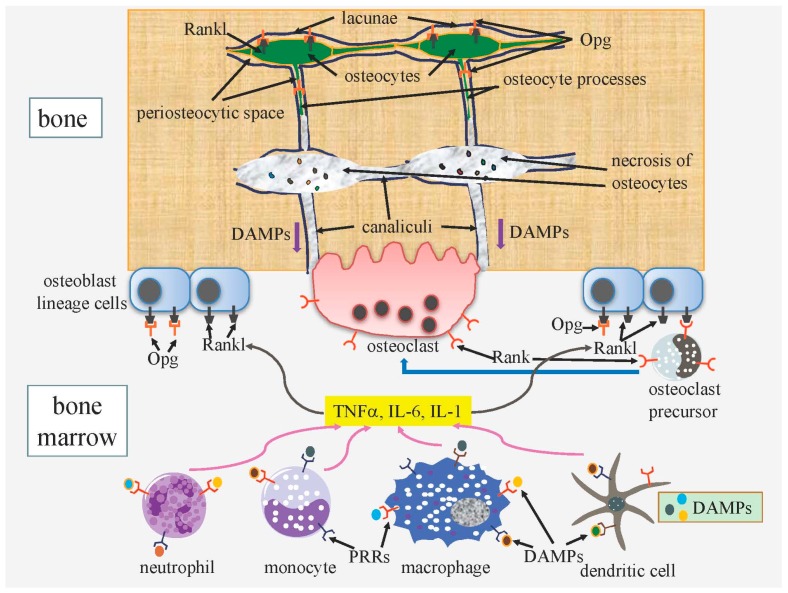
A proposed scheme for the enhanced osteoclastogenesis by osteocyte death. Any type of osteocyte death ultimately results in necrosis because dead osteocytes are not phagocytosed. DAMPs (danger-associated molecular patterns) released from necrotic osteocytes pass through canaliculi and reach the bone surface. Macrophages, dendritic cells, neutrophils, and monocytes are stimulated by DAMPs through PRRs (pattern recognition receptors), and produce TNFα, IL-6, and IL-1, which stimulate the expression of Rankl in osteoblast lineage cells. Osteoclast precursors differentiate into osteoclasts through Rankl-Rank signaling. Opg released from osteocytes to the bone surface negatively regulates osteoclastogenesis and osteoclast activity. This scheme is predicted from previous studies, but has not been directly proven in damaged bone.

Osteocytes, which are embedded in the bone matrix, form a network that is composed of two communication systems in bone. One is an intracellular communication system via gap junction-coupled cell processes, and the other is an extracellular communication system through canaliculi, through which osteocyte processes pass (Figure 3 and Figure 4). Both communication systems are extended to osteoblasts on the bone surface [93]. The extracellular communication system and hemichannels, which are transportation pathways between the processes and canaliculi, are required for the survival of osteocytes, which acquire nutrients, oxygen, and survival signals through them [33,81]. Two junctional proteins—connexins and pannexins—form membrane channels permeable to large molecules in vertebrates [94]. In bone cells, gap junctions are mainly formed by connexin 43 (Gja1), and hemichannels are formed by Gja1 and pannexins. There are three members in the pannexin family: pannexin 1 (Panx1) and pannexin 3 (Panx3) are expressed in bone cells, with Panx1 already being extensively examined [95]. In apoptotic cells, Panx1 undergoes caspase-mediated cleavage at the C terminus for activation, and activated Panx1 channels release adenosine triphosphate (ATP) as a “find me” signal necessary for macrophage recruitment [96]. Extracellular ATP binds to P2Y G-protein-coupled receptors and P2X ligand-gated ion channels—both of which are expressed in osteoblasts, osteocytes, and osteoclasts—and activate them [97]. The activation of P2Y_1_ receptors (P2Y_1_R) enhances the receptor activator of nuclear factor κ-B ligand (Rankl) expression in osteoblasts, the activation of P2Y_6_R in osteoclasts increases their survival, the activation of P2X_7_R in osteoclast precursor cells allows membrane fusion to form multinucleated osteoclasts, and P2X_7_R activates Panx1 for ATP release [95,98,99,100,101] (Figure 3). Since Rankl induces osteoclastogenesis and activates osteoclast functions, the activation of P2YR and P2XR by ATP leads to enhanced bone resorption.

Osteocyte death occurs during aging, after menopause, under unloading, and pathological conditions such as microcracks, and the death of osteocytes is closely coupled to bone resorption [102,103,104]. The microdamage of bone has been associated with osteocyte apoptosis and targeted bone resorption [105,106]. Furthermore, bone resorption is markedly enhanced by the induction of osteocyte death [107]. Cells mainly die through one of three pathways, i.e., apoptosis, autophagic cell death, and necrosis, under physiological and pathological conditions [108]. In osteocyte apoptosis, ATP released from Panx1 channels enhances Rankl expression in the neighboring osteocytes and Rankl up-regulation and bone resorption are attenuated in *Panx1*^−/−^ mice and *P2X_7_R*^−/−^ mice [109,110]. Therefore, ATP released from Panx1 channels, which are activated by P2X_7_R, in apoptotic osteocytes enhances Rankl expression in the neighboring osteocytes and osteoblasts, recruits macrophages, and increases osteoclasts in the neighboring bone surface and vascular canals (Figure 3).

#### 3.1.2. DAMPs Released from Necrotic Osteocytes Further Enhance Bone Resorption

Osteocyte death is distinct because osteocytes are isolated in the bone matrix and apoptosis and autophagic cell death end in secondary necrosis due to the absence of phagocytosis by scavengers [33]. Necrosis leads to the rupture of the cytoplasmic membrane, with most of the intracellular content being released into the extracellular environment [111]. Immunostimulatory molecules including DAMPs, such as S100 family molecules, the HMGB1 protein, purine metabolites, heat-shock proteins, and uric acid, are released through canaliculi to the bone surface and vascular canals in bone (Figure 4). Released DAMPs bind to PRRs, such as TLR2, TLR4, and RAGE, on macrophages, dendritic cells, monocytes, and neutrophils, and promote the production of proinflammatory cytokines including tumor necrosis factor-α (TNF-α), interleukin (IL)-6, and IL-1, which induces the expression of Rankl in osteoblast lineage cells [33,112,113,114,115,116]. Therefore, osteocyte necrosis further enhances Rankl expression and osteoclastogenesis, which have been triggered by ATP released from apoptotic osteocytes, and induced and activated osteoclasts dissolve the neighboring bone surface, leading to the remodeling of damaged bone parts (Figure 4). Since bone resorption after osteocyte apoptosis is attenuated in *Panx1*^−/−^ mice [110], the release of ATP from Panx1 channels appears to be a prerequisite for bone resorption. Although the scheme in Figure 4 for bone resorption by osteocyte necrosis is predicted from previous studies, direct evidence for this scheme has not yet been obtained in damaged bone.

### 3.2. Functions of Osteocytes

#### 3.2.1. Do Live Osteocytes Inhibit Bone Resorption?

A number of mouse models generated in order to elucidate the functions of osteocytes have encountered difficulties due to the death of osteocytes because this process induces bone remodeling as a repair process (Figure 3 and Figure 4). Osteocyte ablation by diphtheria toxin, the deletion of *Gja1* (connexin 43), which constitutes gap junction channels and hemichannels, and osteocyte-specific transgenic mice expressing mutant *Gja1*, which blocks gap junction channels and hemichannels, cause osteocyte apoptosis, leading to secondary necrosis, enhanced bone resorption, and bone remodeling [107,117,118]. The extracellular communication system formed by canaliculi is essential for the bone remodeling process because ATP and the immunostimulatory molecules released from apoptotic cells and necrotic cells, respectively, pass through canaliculi to reach the bone surface or vascular canals (Figure 3 and Figure 4). Most osteocytes die in osteoblast-specific *BCl2* transgenic mice using the 2.3-kb *Col1a1* promoter because the osteocyte processes and canaliculi are severely reduced, possibly due to the formation of a complex of BCL2, actin, and gelsolin, which reduces gelsolin-severing activity in order to increase actin polymerization [81,119]. In *BCl2* transgenic mice, osteocyte death does not induce bone resorption because the extracellular communication system is also disrupted [120]. Osteoclastogenesis and bone resorption are reduced and bone formation is enhanced, leading to an increased bone mass in *BCl2* transgenic mice. These findings indicate that the osteocyte network stimulates osteoclastogenesis and bone resorption and inhibits osteoblastogenesis and bone formation under physiological conditions. Furthermore, these osteocyte functions are augmented under unloaded conditions [33,120].

#### 3.2.2. Regulation of the Release of Osteocyte-Derived Opg to the Bone Surface May Be a Major Role for Rankl on Osteocytes

Osteocytes strongly express Rankl and Opg, which is a secreted decoy receptor of Rankl [120,121,122]. The osteocyte-specific deletion of *Rankl* results in the inhibition of bone resorption, indicating that osteocytes are a major source of Rankl [121,122]. However, the direct interaction of osteocyte processes and osteoclast precursors appears to be limited and the physiological significance of soluble Rankl from osteocytes is unclear. We previously proposed that a major function of Rankl on osteocytes is the regulation of Opg release to the bone surface and vascular canals by trapping Opg [33]. Opg secreted from osteocytes is trapped by Rankl on the surface of osteocytes; however, some Opg is released to the bone surface and vascular canals through canaliculi and binds to Rankl on the surface of osteoblasts, thereby inhibiting osteoclastogenesis and the activation of osteoclasts [33]. This needs to be proved through the generation of osteocyte-specific *Opg*/*Rankl* double knockout mice. In the region with dead osteocytes, the release of Opg to the bone surface through canaliculi will be reduced by the up-regulated expression of Rankl in the neighboring osteocytes. The reduced release of Opg will further enhance osteoclastogenesis and osteoclast activity (Figure 3 and Figure 4).

## 4. Conclusions

Chondrocyte proliferation is strictly regulated in vivo and acceleration of the cell cycle results in p53-dependent apoptosis. Although the inhibition of osteoblast apoptosis increases bone mass, the effects of apoptosis on bone formation are largely dependent on p53, which exerts opposite effects on bone formation by inhibiting the proliferation of and enhancing apoptosis in osteoblasts and also by accelerating osteoblast differentiation through the Akt–FoxOs pathway. Chondrocyte death is involved in the onset and progression of posttraumatic OA. Osteocyte apoptosis is predicted to enhance bone resorption through the release of ATP, and subsequent osteocyte necrosis is predicted to enhance bone resorption through the release of DAMPs, leading to the replacement of damaged bone.
